# Extracorporeal membrane oxygenation for main airway rupture secondary to severe thoracic trauma: A case report

**DOI:** 10.1016/j.cjtee.2025.09.004

**Published:** 2026-05-21

**Authors:** Shuhong Wang, Yin Tian, Xiangui Zhou, Peng Wang, Zhiheng Jiang, Ruilie Wang, Changqiang Liang, Guang Yang, Peng Ye, Anyong Yu, Tinggang Wang

**Affiliations:** a Department of Emergency, Affiliated Hospital of Zunyi Medical University, Zunyi, 563000, Guizhou, China; b Department of Cardiology, The First People's Hospital of Zunyi, Zunyi, 133012, Guizhou, China

**Keywords:** Extracporeal membrane oxygenation (ECMO), Main airway rupture, Severe thoracic trauma, Airway repair

## Abstract

Thoracic injuries due to severe trauma are common, but major airway rupture is a rare and fatal complication. When a patient suddenly loses effective ventilation, extracorporeal membrane oxygenation (ECMO) is an effective rescue method to ensure gas exchange. ECMO can play an important role for patients with severe airway injury who do not respond to conventional treatments. This article reports a rare case of thoracic and abdominal injuries. A 28-year-old freight worker fell from a height of approximately 20 m along with a car. He sustained severe trauma to the chest, and oxygen saturation could not be maintained. Chest CT scan and reconstruction showed bilateral pneumothorax, extensive subcutaneous emphysema in the neck, bilateral chest wall, chest, and back, pulmonary contusion, and multiple rib fractures. An effective multidisciplinary team cooperated to urgently perform ECMO life support and completed airway repair surgery and laparotomy exploration. The patient ultimately achieved a good functional prognosis. After 35 days of recovery, the patient was discharged from the hospital. After 1 to 6 months of follow-up after discharge, his recovery was good. This study provides treatment experience for patients with traumatic main bronchial rupture and may help improve the success rate of treatment for such patients.

## 1 Introduction

Patients with clinical trauma are those who have suffered damage to body tissues or organs due to various mechanical factors and require evaluation, diagnosis, and treatment in a clinical setting (e.g., hospital emergency room, trauma center, etc.).^1^ With the rapid development of society, the occurrence of high-energy injuries dominated by traffic accidents, collapse accidents, and crush accidents has become more and more frequent, resulting in a high incidence of severe multiple injuries.^2^ Even in economically developed countries with relatively well-developed medical and emergency systems, trauma remains the leading cause of death among people under 45 years of age.^3^ The older population is the primary impact of traumatic events.^4^ In China, trauma deaths have accounted for 10% of annual deaths in recent years, and their mortality and disability rates are significantly higher than those in developed countries in Europe and the United States, making trauma the 5th leading cause of death in China, with obvious epidemiological characteristics.^5^, ^6^, ^7^, ^8^ Thoracic trauma can be severe, and most cases of thoracic trauma with multiple rib fractures are accompanied by intrathoracic hemorrhage and pneumothorax due to intercostal vascular injury, which makes treatment difficult.^9^ Among them, rib fractures are the most prevalent type, accounting for the majority of thoracic trauma.^10^ Patients with traumatic rib fractures are often treated with thoracoscopy-assisted rib fracture fixation.^11^ Penetrating cardiac trauma is an extremely severe thoracic trauma with an extremely high mortality rate.^12^

Main tracheal rupture following thoracic trauma is a rare and fatal event.^13^ In 2025, Leivaditis et al.^12^ reported a case of spiral rupture of the right main bronchus following a motorcycle accident; the patient underwent pneumonectomy and was discharged in good health 15 days postoperatively. With the development of society, the number of traffic accident cases is increasing year by year, and the number of patients with thoracic trauma is increasing. Accurate diagnosis and treatment can reduce the case fatality rate to 9%.^13^ Treatment of these patients often requires aggressive surgical intervention to relieve airway obstruction and restore thoracic stability. There is also endoscopically assisted endotracheal intubation, in which the catheter passes through the site of injury to supply air to the distal lung tissue. However, the premise of surgery is that relatively stable vital signs are required to tolerate the surgery. Extracorporeal membrane oxygenation (ECMO) is a medical emergency device and technique used primarily to provide continuous extracorporeal respiratory and circulatory support to patients with severe cardiopulmonary failure, thereby maintaining the body's circulation and gas exchange.^14^^,^^15^ The use of ECMO for cardiopulmonary support in adults with traumatic tracheobronchial injury has been reported.^16^ In this case, the patient had undergone endotracheal intubation before surgery, but SpO_2_ was always difficult to maintain with ventilator support, and tracheal rupture was confirmed by preoperative flexible bronchoscopy. This case represents an extremely rare form of combined trauma, making its assessment, treatment, and care management very challenging.

## 2 Case report

### 2.1 Time frame

The key clinical timeline for this patient's management is as follows (Fig. 1). On admission (15:10), initial vital signs revealed tachycardia (150 beats/min) and hypotension (110/53 mmHg). At 15:14, blood pressure had dropped to 80/48 mmHg, indicating severe hypovolemia. Dual-channel fluid resuscitation was initiated immediately, along with the transfusion of 2 units of O-type red blood cells, followed by the insertion of a central venous catheter at 15:19. At 15:30, vital signs had stabilized slightly (heart rate 140 beats/min, blood pressure 80/52 mmHg). A bedside bronchoscopy (15:50–16:20) revealed a laceration of the left main bronchus (Fig. 2). At 16:20, preoperative investigations were conducted (Fig. 3). The patient entered the operating theatre at 16:40 and was placed on ECMO support at 17:00 (heart rate 147 beats/min, blood pressure 85/52 mmHg) (Fig. 4). At 17:30, open thoracotomy combined with thoracoscopic repair of the bronchial injury was performed, followed by exploratory laparotomy. Postoperatively, vital signs improved, and the patient was transferred to the intensive care unit (ICU) at 22:30. The patient recovered well and was discharged walking 35 days after surgery.

**Fig. 1:**
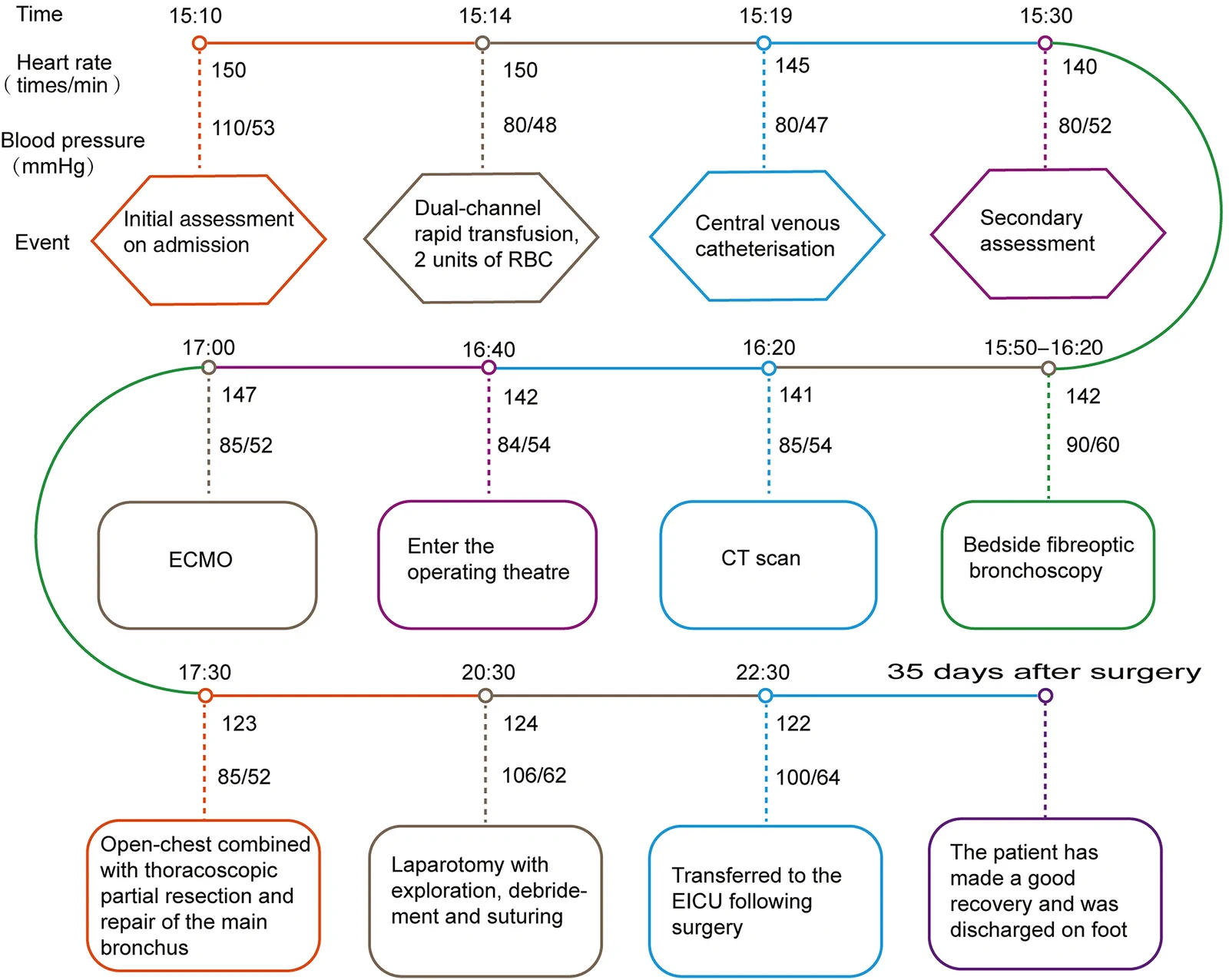
Key time points before and after the patient's admission.

**Fig. 2:**
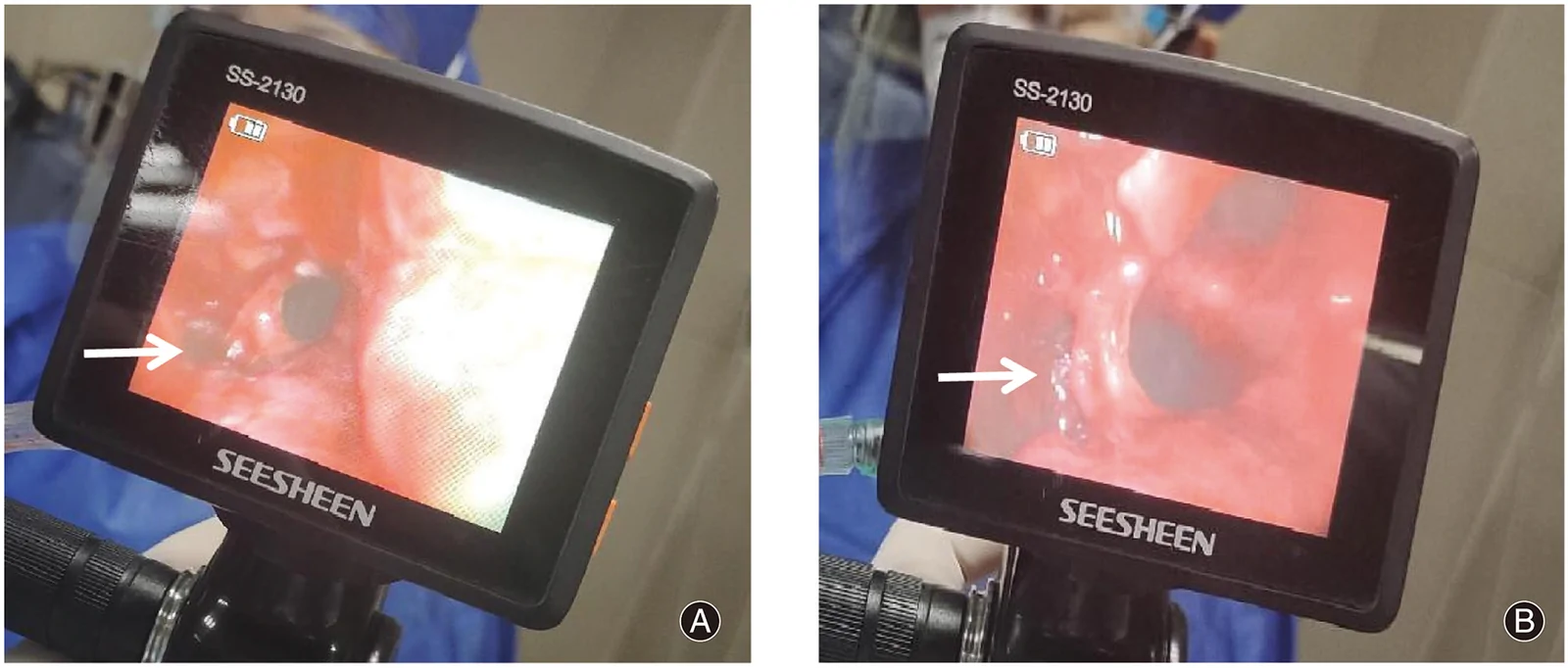
Fibrobronchoscopy images of a patient with a ruptured bronchus. (A) and (B) show a rupture in the right bronchus.

**Fig. 3:**
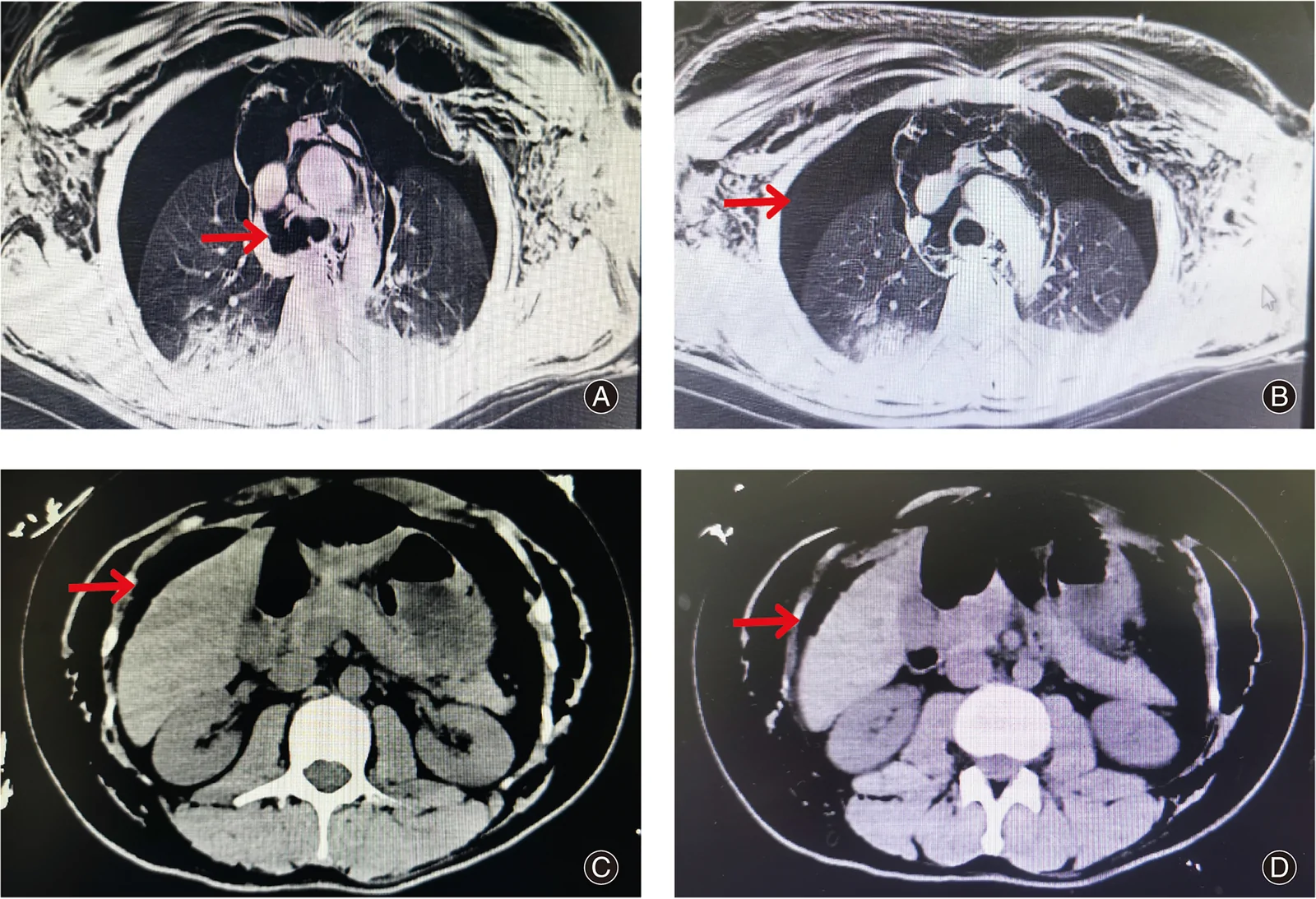
(A) Rupture of the right main bronchus. (B) Widerspread subcutaneous emphysema and a large pneumothorax. (C & D) Extensive pneumoperitoneum on abdominal CT.

**Fig. 4:**
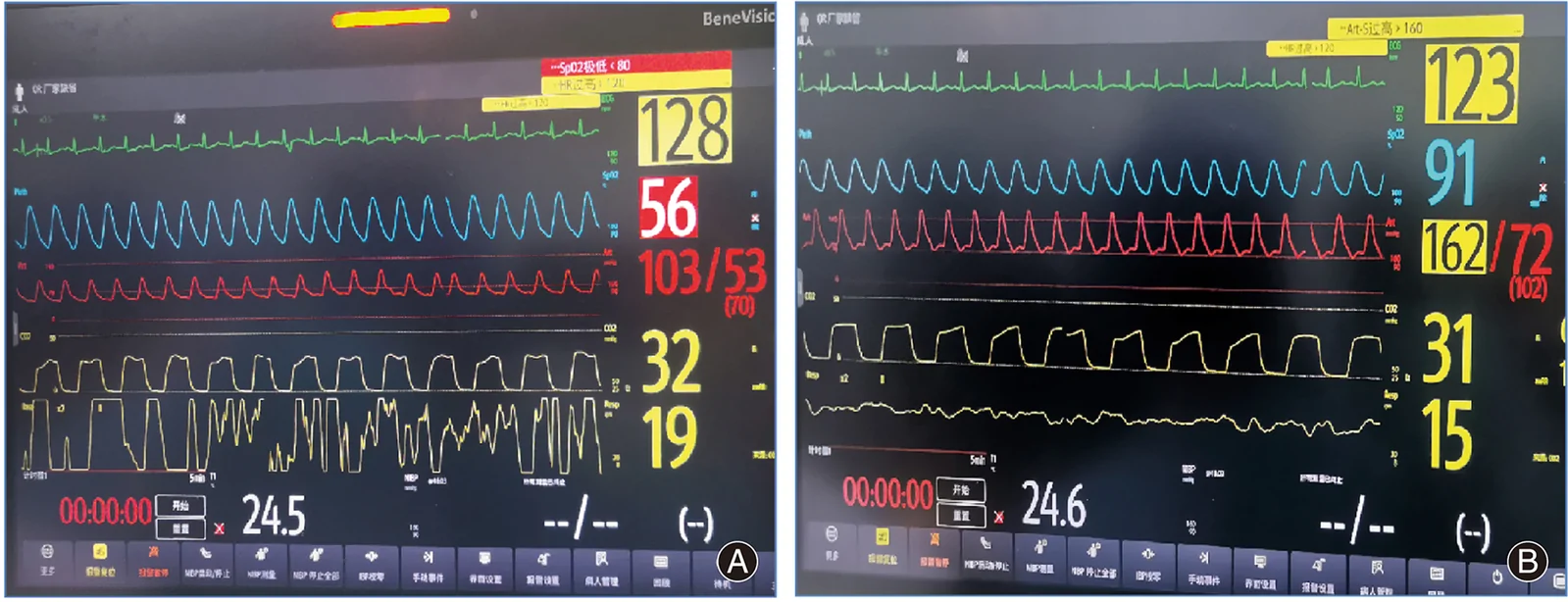
(A) The graph shows the patient's vital signs and oxygen saturation levels before ECMO treatment. (B) The graph shows the patient's vital signs and oxygen saturation levels following ECMO treatment. ECMO: extracorporeal membrane oxygenation.

### 2.2 Case study

A 28-year-old male freight worker was taken by ambulance to a local emergency room. The patient was almost unconscious, depressed, and had a pained expression, but was able to answer questions accurately. The local hospital performed endotracheal intubation with an invasive ventilator to assist breathing, bilateral thoracostomy, and other treatments, after which he was referred to our hospital.

His vital signs upon admission to the emergency room were as follows: body temperature 37.5 °C, heart rate 155 beats/min, respiratory rate 20 beats/min (breathing with respiratory sac assistance), blood pressure 110/53 mmHg (mintained with vasoactive drugs). The patient denies any history of chronic or infectious diseases and has no previous history of trauma or surgery. His history of blood transfusion or genetic history was unknown.

The patient's pupils were symmetrical, approximately 3 mm in diameter, round in shape, and reactive to light. Blood was visible in the nasal cavity and oral cavity. A rebar has penetrated the oral cavity. Bilateral breath sounds are not clearly audible upon auscultation. No obvious limb abnormalities are observed. Skin ecchymosis is visible on the right upper limb. Muscle strength in the upper limbs is grade V, with no abnormality in the lower limbs and no muscle tone disorder. The dorsalis pedis pulse on the right foot is not significant, while it is normal on the left foot. Bilateral Babinski reflexes are negative. The injury severity score was 30 points. It is the first injury severity assessment method based on the abbreviated injury scale, covering 6 areas: head and neck, face, chest, abdominal and pelvic organs, extremities and pelvis, and skin. The scores range from 1 to 75 points, with <16 indicating minor injury, 16–25 indicating severe injury, and ≥25 indicating critical injury. Emergency head and whole-body CT reconstruction revealed the following: brain contusion, skull and skull base fractures, subarachnoid hemorrhage, maxillary injury, tongue injury, right thyroid lobe injury, pleural effusion, lung contusion, cardiac contusion, left lung lobe injury, neck and mediastinal emphysema, and pneumothorax. Due to the patient's critical condition and unstable oxygen saturation, 3-dimensional bronchial reconstruction was not performed. The diagnosis of left main bronchial rupture was confirmed by bedside flexible bronchoscopy, which provided real-time visualization of the injury site. The main diagnosis was penetrating trauma, including severe open cranial injury, multiple brain contusions, traumatic subarachnoid hemorrhage, skull fracture, skull base fracture, cerebrospinal fluid leakage, oral penetrating injury, palate injury, tongue injury, right thyroid lobe injury, neck penetrating injury, chest penetrating injury, right pneumothorax, pleural effusion, lung contusion, cardiac contusion, left hepatic lobe penetrating injury, diaphragm penetrating injury, abdominal wall penetrating injury, scrotal penetrating injury, and right upper limb soft tissue contusion.

### 2.3 Emergency care

Following emergency admission, the critically injured patient received standardized emergency treatment focusing on airway protection, respiratory support, circulatory resuscitation, and basic emergency care. Given the patient's severe tracheobronchial lacerations and refractory respiratory distress, emergency tracheal intubation was performed immediately to maintain airway patency, followed by invasive mechanical ventilation to correct severe hypoxemia. To alleviate pneumothorax and extensive subcutaneous emphysema, bilateral closed chest drains were inserted. All drains were securely fixed to prevent kinking, compression, or accidental dislodgement, and the volume, colour, and nature of the drainage were closely monitored and dynamically recorded to reflect real-time changes within the pleural cavity. Dual intravenous access was rapidly established, and aggressive fluid resuscitation and blood transfusion were administered to stabilize hemodynamics and reverse hypovolemic shock. Throughout the emergency management, vital signs, including heart rate, blood pressure, and oxygen saturation, were continuously monitored. Rigorous airway management was implemented, comprising standardized sterile suctioning of secretions and routine oral care, with ventilator settings adjusted promptly based on arterial blood gas analysis results to prevent complications such as atelectasis and secondary pulmonary infection. As a precautionary measure, a cervical collar was fitted to the patient and removed immediately once cervical spine fracture had been ruled out. As a precautionary measure, a cervical collar was fitted to the patient and removed immediately once cervical spine fracture had been ruled out. All resuscitation equipment and medication were kept within easy reach so that a rapid response could be mounted should the patient's condition deteriorate suddenly. Following comprehensive resuscitation and the stabilization of the patient's condition, preparations were made for definitive surgical repair.

### 2.4 Clinical treatment

After the patient arrived at our hospital for basic emergency care and examination, it was recommended to undergo thoracic combined thoracoscopy, right main bronchiectomy, right main bronchial repair, and right tracheal carina plasty. At the same time, the patient had undergone tracheal intubation before surgery; however, SpO_2_ was always difficult to maintain with ventilator support, with heart rate remaining high and the blood pressure continuously dropping, which was confirmed as a tracheal rupture preoperative flexible bronchoscopy. Therefore, preoperative ECMO supportive care is recommended. Due to severe lung injury, main bronchial rupture combined with massive pneumothorax (predominantly on the left side), ECMO could not completely reverse the patient's hypoxemia. In this case, the patient had severe shock and respiratory failure caused by bronchial rupture after trauma, and the ECMO used was in veno-venous ECMO (V-VECMO) mode, in which blood was drawn out of a vein and, after membrane oxygenation, pumped back to a vein. This mode can replace some functions of the heart and lungs simultaneously, and maintain oxygenated blood perfusion and circulatory power throughout the body. In V-VECMO mode, the femoral vein and internal jugular vein were selected as the puncture sites. At each puncture site, local anesthesia was first administered, followed by puncture and catheterization of the femoral vein and internal jugular vein using the Seldinger technique (percutaneous puncture insertion of the guidewire, followed by insertion of the catheter along the guidewire). Ultrasound guidance is used to ensure that the catheter is positioned accurately during catheter placement, thereby helping to avoid inadvertent entry into arteries or other blood vessels. The catheters that are used for drainage and return are then connected to the ECMO line, respectively. The pipeline is pre-filled before connection, typically with normal saline, albumin, or other liquids to remove air from the pipeline and prevent air embolism. During the pre-filling process, it is essential to ensure that there is no liquid leakage and no bubble residue in the pipeline system. When the tubing is properly connected and pre-filled, the blood pump is activated and begins to drain the patient's venous blood. Closely observe the patient's blood oxygen saturation, blood gas analysis results, heart rate, blood pressure and other indicators, and adjust the ECMO settings accordingly.

After maintaining the blood flow rate and oxygenation with the support of ECMO, the patient was sent to the operating room. ECMO was used to replace intraoperative ventilator support, and pulmonary ventilation was terminated. A thoracotomy combined with thoracoscopy, right main bronchiectomy, right main bronchial repair, and right tracheal carina was then performed. The right posterolateral chest incision was selected. The skin, muscle, and other tissues were cut layer by layer, and the thoracic cavity was entered through the intercostal space or after partial rib resection, to fully expose the surgical field and explore the overall situation in the thoracic cavity. During the operation, the distal trachea was examined, and the rupture site in the right main bronchus and the extent of surrounding tissue damage were identified. Considering direct suture having resulted in high tension, the right main bronchus was dissociated to release the distal end, and both the left and right main bronchi were freed toward both sides. At the same time, the severed end of the proximal trachea was freed to the level of the innominate vein. During the operation, the tissues around the right main bronchus, including loose connective tissue and blood vessels, were carefully dissected to avoid damage to important vascular structures such as pulmonary arteries and pulmonary vein branches. Meanwhile, the surrounding nerve tissue was protected, and adequate mobilization of the ruptured right main bronchus was ensured for subsequent surgical operations. In this case, the patient had severe damage at the fracture site with lesion tissue that was difficult to recover, so the right main bronchus was excised 0.5 cm from the edge of the lesion, so that the area of the membranous defect below the broken end was the same as the excess tissue above the broken end, and the fracture margin was trimmed. During the resection process, over-resection was avoided to prevent difficulty in subsequent anastomosis or impairment of lung function, and the blood vessels of the bronchial stump were properly managed after resection to prevent bleeding. The right main bronchial repair was then performed to repair the stump after removing part of the bronchi. Continuous suturing was performed from the left posterior wall of the trachea using absorbable sutures to ensure bronchial integrity and tightness. When suturing, attention was paid to adjusting the suture tension and ensuring uniform stitch distance and margin, so that the tissues were fully aligned, avoiding the formation of anastomotic narrowing or leakage caused by sutures that were too tight or too loose, and ensuring the patency of the bronchial lumen after repair. In this case, the bronchial rupture was close to the tracheal carina, affected the structure and function of the tracheal carina, and required right tracheal carina plasty. Appropriate trimming and displacement of the right main bronchus were performed, and the right main bronchus was reconnected with the trachea to restore normal airway structure and airflow path. During the connection process, the angle and tension of the connection port were adjusted to ensure that the connected airway was unobstructed and stable. The multi-needle interrupted suture technique was used to carefully align the mucosal layer, muscle layer, and other structures of the trachea and bronchi. After the completion of the plasty, the lungs were inflated by injecting normal saline into the airway, and the anastomosis was checked for air leakage. The shape of the tracheal carina, bronchi, and the patency of the lumen, were carefully observed to ensure that the surgical effect met expectations. The chest cavity was checked for bleeding spots, and hemostasis was performed at any possible sites. After the suture was completed, a thoracostomy drainage tube was placed appropriately in the thoracic cavity to drain postoperative intrapleural blood and air, thereby promoting lung recruitment and restoring normal thoracic physiology. Then, the thoracic incision was closed layer by layer, including suturing muscles, fascia, subcutaneous tissues, and skin, with attention to suture quality at each layer to avoid postoperative incision-related complications. Finally, laparotomy, debridement, and suture were performed. After all surgeries, the patient was transferred back to the ICU with V-VECMO retained.

Upon admission to the intensive care unit, systemic heparin anticoagulation was initiated immediately, with activated clotting time maintained at 160–180 sec and activated partial thromboplastin time controlled at 45–60 sec. Heparin dosage was dynamically adjusted based on D-dimer levels and thromboelastography. Postoperative management included concurrent administration of analgesia, sedation, haemostasis, anti-infective therapy, and comprehensive symptomatic supportive care. During the ICU stay, a management strategy prioritising analgesia combined with early sedation was adopted, effectively reducing oxygen consumption and improving tissue perfusion and ventilation. Once the patient's haemodynamics had stabilized, the dose of sedative medication was gradually reduced. For antimicrobial therapy, empirical treatment with cefotaxime was administered in accordance with the characteristics of the trauma, with the antibiotic regimen subsequently adjusted based on the results of blood and bronchoalveolar lavage fluid cultures. On postoperative day 5, the patient's condition improved markedly. With ECMO operating at a rotational speed of 224 r/min, a blood flow rate of 2.2 L/min, and an oxygen flow rate of 1.2 L/min, the patient's oxygen saturation remained consistently above 95% and circulation was stable, and then the ECMO was successfully weaned. A follow-up chest CT scan on postoperative day 21 showed good restoration of tracheal continuity, complete resolution of the pneumothorax, and adequate re-expansion of the right lung, whilst atelectasis persisted in the left lung, and a follow-up chest X-ray on postoperative day 25 demonstrated complete re-expansion of the right lung and partial re-expansion of the left lung. The patient's overall recovery was uneventful, and on postoperative day 35, he was able to get out of bed unaided and was discharged following full recovery. Throughout the perioperative period, a multidisciplinary collaborative care model was adopted to tailor a personalized, accurate, and comprehensive diagnosis and treatment plan, thereby maximizing treatment effect, improving prognosis, and enhancing the quality and safety of medical care.

## 3 Discussion

In trauma patients, bronchial injuries are relatively rare due to the protective effect of thoracic and mediastinal tissues on the bronchi, and most rupture sites are within 2.5 cm of the carina, resulting in a mortality rate of up to 30%.^17^ When the bronchi are ruptured, free air can enter the mediastinum and pleural space, leading to the formation of a pneumothorax. Approximately 70% of patients with bronchial rupture will have a concomitant pneumothorax.^18^ Bronchial injuries can be classified into 2 types based on clinical symptoms. Type I is characterized by the disconnection of the trachea and bronchi from the thoracic cavity, with hemopneumothorax as the main symptom after rupture. Type II is characterized by the severed ends of the trachea and bronchi within the mediastinum, with mediastinal emphysema as the main symptom. This case was classified as Type I. Based on the time elapsed between injury and treatment, bronchial injuries can be classified as early-stage (treated within 1 week), mid-stage (between 1 week and 1 month), and late-stage (more than 1 month). In this case, the patient was admitted for treatment promptly following the injury, which contributed to a higher success rate in clinical management. In the diagnosis of bronchial injury, the diagnosis is mainly confirmed by X-ray, CT, and bronchoscopy. X-rays can show emphysema, pneumothorax, and gas shadows in the trachea and peribronchial tubes, presenting with "weeping willow disease".^19^^,^^20^ CT scans can accurately determine the exact location of bronchial injury.^21^ In this case, CT scan not only helped accurately determine the location of the bronchial injury, providing intuitive anatomical information essential for surgical planning and risk assessment, but also allowed monitoring of patient's condition and treatment effect through CT images. The patient in this case presented with multiple injuries and had a poor tolerance for extensive surgery. Consequently, clinical management prioritised the treatment of life-threatening tracheal injuries. However, due to the continuous nature of the airway rupture, conventional tracheal tubes were unable to traverse the rupture, presenting significant challenges in establishing an artificial airway.^22^ Fibreoptic bronchoscopy can effectively assist in managing difficult airways, as it allows for direct and visual assessment of the extent and severity of airway injury, whilst providing precise guidance for tracheal intubation, and ensuring accurate placement of the artificial airway. At the same time, it reduces the need for blind procedures within the airway, thereby lowering the risk of associated complications. Furthermore, airway reconstruction surgery requires adequate exposure of the surgical field to facilitate precise reconstruction, and relying solely on conventional endotracheal intubation makes it difficult to maintain effective intraoperative oxygenation, as it fails to meet the oxygen supply requirements of complex airway reconstruction procedures.^23^

ECMO is a vital form of extracorporeal life support that effectively takes over the functions of the heart and lungs, maintains circulatory stability, improves oxygenation and carbon dioxide clearance, and reduces the burden on damaged cardiac and pulmonary tissues. It is now widely used in the treatment of patients with severe cardiopulmonary injury. In recent years, the value of ECMO in the management of severe trauma, particularly complex airway injuries, has become increasingly apparent. Traumatic tracheal and bronchial ruptures are life-threatening conditions; conventional mechanical ventilation often fails to maintain effective oxygenation, and conventional surgical procedures carry extremely high risks.^23^^,^^24^ Our patient presented with a blunt penetrating chest injury complicated by a ruptured right bronchus. Following intubation, the patient developed refractory hypoxemia and was unable to tolerate emergency surgery. After urgent initiation of ECMO support, a combined airway repair and exploratory laparotomy was successfully performed. Multiple clinical studies have demonstrated that ECMO can provide stable respiratory support during surgery for patients with traumatic airway rupture, thereby mitigating fatal risks such as hypoxia and asphyxia, and creating optimal conditions for precise airway reconstruction. ECMO can fully compensate for impaired pulmonary gas exchange, alleviating intraoperative oxygenation constraints and facilitating surgical repair of complex airway injuries. Early and appropriate use of ECMO support has become a key strategy in the multidisciplinary management of trauma complicated by severe airway injury. It effectively enhances surgical safety and treatment success rates, offering significant clinical value in the management of severe trauma.

The successful treatment of this case highlighted the important role of flexible bronchoscopy in the establishment and the great potential of ECMO in the treatment of traumatic patients with ruptured main airway. Through multidisciplinary cooperation and the comprehensive use of various advanced medical technologies, the treatment success rate of patients with severe trauma can be significantly improved. In the future, continuous advances in medical technology and the accumulation of case experience will enable more accurate and efficient treatment of such complex cases.

## CRediT authorship contribution statement

**Shuhong Wang:** Writing – original draft. **Yin Tian:** Writing – original draft. **Xiangui Zhou:** Data curation. **Peng Wang:** Data curation. **Zhiheng Jiang:** Data curation. **Ruilie Wang:** Data curation. **Changqiang Liang:** Data curation. **Guang Yang:** Data curation. **Peng Ye:** Data curation. **Anyong Yu:** Data curation. **Tinggang Wang:** Writing – review & editing.

## Ethical statement

The manuscript has been approved by the appropriate ethical committees related to the institution in which it was performed (KLL-2024-747).

## Funding

This research was supported by grants obtained from the 10.13039/501100001809National Natural Science Foundation of China (grant number 82260254), Zunyi Science and Technology Project (no. Zunshi Kehe HZ 2023218) and Peking Union Medical College RuiE Emergency Medicine Research Fund PUMF01010010-2024-23.

## Declaration of competing interest

The authors declare that they have no competing interests.

## References

[1] Wang M., Wang S., Wang Y. (2024). Bibliometric and visual analysis of traumatic cardiac arrest. Chin J Emerg Med.

[2] Rosen H.E., Bari I., Paichadze N. (2025). Global road safety 2010-18: an analysis of global Status Reports. Injury.

[3] Benhamed A., Batomen B., Boucher V. (2023). Epidemiology, injury pattern and outcome of older trauma patients: a 15-year study of level-I trauma centers. PLoS One.

[4] Jojczuk M., Pawlikowski J., Kamiński P. (2023). Evaluating changes in trauma epidemiology during the COVID-19 lockdown: insights and implications for public health and disaster preparedness. Healthcare (Basel).

[5] Zhang L.Y., Zhang X.Z., Bai X.J. (2018). Current trauma care system and trauma care training in China. Chin J Traumatol (Engl Ed).

[6] González L.R., Riquelme U.A., Fuentes E.A. (2018). [Thoracic trauma. Experience of three decades]. Rev Med Chile.

[7] Hajjar W.M., Al-Nassar S.A., Almutair O.S. (2021). Chest Trauma experience: incidence, associated factors, and outcomes among patients in Saudi Arabia. Pakistan J Med Sci.

[8] Sarmış N.B., Kuzucuoğlu M., Taylan K.İ. (2024). The effect of thoracic trauma scoring system in thoracic trauma patients with rib fracture. Acta Chir Belg.

[9] Wang D., Xu Y., Wang Q. (2022). A cohort Study on the comparison of complications, short-term efficacy, and quality of life between thoracoscopic surgery and traditional surgery in the treatment of rib fractures. Contrast Media Mol Imaging.

[10] González-Hadad A., Ordoñez C.A., Parra M.W. (2021). Damage control in penetrating cardiac trauma. Colomb Méd.

[11] Karmy-Jones R., Wood D.E. (2007). Traumatic injury to the trachea and bronchus. Thorac Surg Clin.

[12] Leivaditis V., Karamouzos V., Aretha D.N. (2025). Spiral bronchial rupture in a young trauma patient: critical role of bronchoscopy and surgical precision. Cureus.

[13] Díaz C., Carvajal D.F., Morales E.I. (2019). Right main bronchus rupture associated with blunt chest trauma: a case report. Int J Emerg Med.

[14] Na S.J., Jeon K. (2020). Extracorporeal membrane oxygenation support in adult patients with acute respiratory distress syndrome. Expert Rev Respir Med.

[15] Ko R.E., Chung C.R., Yang J.H. (2021). Use of extracorporeal membrane oxygenation in postpartum patients with refractory shock or respiratory failure. Sci Rep.

[16] Walker J.L., Wiersch J., Benson C. (2012). The successful use of cardiopulmonary support for a transected bronchus. Perfusion.

[17] Taskinen S.O., Salo J.A., Halttunen P.E. (1989). Tracheobronchial rupture due to blunt chest trauma: a follow-up study. Ann Thorac Surg.

[18] Altinok T., Can A. (2014). Management of tracheobronchial injuries. Eurasian J Med.

[19] Tao Z., Xu D., Wan Z. (2024). Severe tracheal tear due to endotracheal intubation: a case report. J Thorac Dis.

[20] Gill T.K., Loo G.H., Muthkumaran G. (2025). Posterior membranous tracheal injury during mckeown oesophagectomy. A case report with literature review. Front Oncol.

[21] Ge J.C., Shang M., Yao M.Y. (2024). Application of MSCT image post-processing techniques in the identification of rib Fracture malunion. Fa Yi Xue Za Zhi.

[22] Huo M., Guo C., Zhu G. (2024). [The clinical treatment of laryngotracheal rupture injury]. Lin Chuang Er Bi Yan Hou Tou Jing Wai Ke Za Zhi.

[23] Combes A., Schmidt M., Hodgson C.L. (2020). Extracorporeal life support for adults with acute respiratory distress syndrome. Intensive Care Med.

[24] Martins Lima P., Pinto S.G., Dias J. (2023). The challenges of spinal surgery requiring ventral decubitus in the critical trauma patient on extracorporeal membrane oxygenation: a case report. Cureus.

